# Supplementary material for: House sparrows do not exhibit a preference for the scent of potential partners with different MHC-I diversity and genetic distances
Source: PLoS One. 2022 Dec 21;17(12):e0278892. doi: 10.1371/journal.pone.0278892 (PMC9770374; doi:10.1371/journal.pone.0278892)
Supplement: S1 Table — (DOCX) [file pone.0278892.s001.docx]

**S1. Table.** Data obtained in the behavioural experiment. In the choice: 0 = the focal bird did not choose the most diverse or dissimilar conspecific, 1 = the focal bird chose the most diverse or dissimilar conspecific. Empty cases means that there were not differences in the allele numbers between both scent donor birds.

| **Individual** | **Sex** | **more allelic distance choice** | **more functional distance choice** | **more allelic diversity choice** | **more functional diversity choice** | **knocking** |
| --- | --- | --- | --- | --- | --- | --- |
| 1h | female | 0 | 0 | 1 | 1 | no |
| 2m | male | 1 | 1 | 1 | 1 | yes |
| 3m | male | 1 | 1 | 1 | 1 | yes |
| 4h | female | 0 | 0 | 1 | 1 | yes |
| 4m | male | 0 | 0 |  |  | yes |
| 5h | female | 1 | 1 | 1 | 1 | no |
| 7h | female | 1 | 1 | 0 | 0 | no |
| 7m | male | 0 | 0 | 0 | 0 | yes |
| 8h | female | 0 | 0 | 0 | 0 | no |
| 8m | male | 0 | 0 | 1 | 1 | yes |
| 9h | female | 0 | 0 | 0 |  | no |
| 9m | male | 0 | 0 | 0 | 0 | yes |
| 10h | female | 0 | 1 | 1 | 1 | no |
| 10m | male | 0 | 0 | 0 | 0 | yes |
| 11h | female | 0 | 0 | 1 | 1 | no |
| 11m | male | 0 | 0 |  |  | yes |
| 12h | female | 1 | 1 |  |  | no |
| 13h | female | 0 | 0 | 0 | 0 | yes |
| 14h | female | 0 | 0 |  |  | no |
| 14m | male | 1 | 1 |  | 1 | no |
| 15h | female | 0 | 0 |  |  | no |
| 16h | female | 0 | 1 | 0 | 0 | no |
| 16m | male | 1 | 1 | 0 | 0 | no |
| 17h | female | 0 | 0 | 1 | 1 | yes |
| 17m | male | 1 | 1 | 0 | 0 | yes |
| 18m | male | 0 | 1 | 0 | 0 | no |
| 19h | female | 0 | 0 | 1 | 1 | no |
| 19m | male | 0 | 1 | 1 | 1 | yes |
| 20h | female | 1 | 1 | 0 | 0 | yes |
| 20m | male | 1 | 1 | 0 | 0 | yes |
| 21h | female | 0 | 1 | 0 | 0 | no |
| 21m | male | 1 | 0 | 1 | 1 | yes |
| 22h | female | 0 | 0 |  |  | yes |
| 22m | male | 0 | 0 | 1 | 1 | no |
| 23h | female | 0 | 0 |  |  | yes |
| 24h | female | 0 | 0 | 0 | 0 | no |
| 25h | female | 0 | 0 |  | 1 | no |
| 26h | female | 1 | 0 |  |  | no |
| 26m | male | 1 | 1 | 0 | 0 | no |
| 27h | female | 1 | 1 |  |  | no |
| 28h | female | 0 | 0 | 1 | 1 | no |
| 28m | male | 0 | 0 | 1 | 1 | no |
| 29h | female | 1 | 1 | 0 | 0 | no |
| 30h | female | 1 | 1 | 0 | 0 | yes |
| 30m | male | 0 | 0 | 0 | 0 | no |
| 31h | female | 1 | 1 | 0 | 0 | no |
| 31m | male | 1 | 1 | 0 | 0 | no |
| 32h | female | 1 | 1 |  |  | no |
| 32m | male | 0 | 0 | 1 | 1 | yes |
| 33h | female | 0 | 0 | 0 |  | yes |
| 34h | female | 1 | 1 | 0 | 0 | no |
| 34m | male | 0 | 0 | 0 | 0 | no |
| 35h | female | 0 | 0 | 1 | 1 | no |
| 36m | male | 0 | 0 | 1 | 1 | no |
| 37h | female | 1 | 1 | 1 | 1 | no |
| 38h | female | 1 | 1 | 1 | 1 | no |
| 39h | female | 0 | 0 |  |  | no |
| 40h | female | 0 | 0 | 1 | 1 | no |
| 41h | female | 1 | 1 | 0 | 0 | no |
| 41m | male | 1 | 1 | 0 | 0 | yes |
| 43h | female | 1 | 1 |  | 0 | no |
| 43m | male | 0 | 1 |  | 0 | yes |
| 44h | female | 1 | 1 |  |  | no |
| 44m | male | 0 | 1 |  |  | no |
| 45h | female | 0 | 0 | 0 | 0 | no |
| 45m | male | 1 | 1 | 1 | 1 | yes |
| 46h | female | 0 | 0 | 1 | 1 | no |
| 46m | male | 0 | 1 | 1 | 1 | no |
| 47h | female | 0 | 0 | 1 | 1 | yes |
| 47m | male | 0 | 0 | 0 | 0 | no |
| 48h | female | 1 | 0 | 0 | 1 | no |
| 48m | male | 0 | 0 |  |  | no |
| 49h | female | 0 | 0 | 1 | 1 | no |
| 50h | female | 0 | 0 | 1 | 1 | no |
| 50m | male | 0 | 0 | 1 | 0 | no |
| 51h | female | 1 | 1 | 0 | 1 | no |
| 52h | female | 1 | 1 | 0 | 0 | no |
| 52m | male | 0 | 0 | 0 | 0 | no |
| 53h | female | 1 | 1 | 0 | 0 | yes |
| 53m | male | 1 | 1 | 1 | 1 | no |
| 54m | male | 0 | 0 | 0 | 0 | no |
| 55m | male | 1 | 1 | 0 | 0 | no |
| 57m | male | 1 | 0 | 0 | 0 | no |
| 58m | male | 0 | 0 | 0 |  | no |
| 60m | male | 1 | 1 | 0 | 0 | no |
| 61m | male | 1 | 1 |  |  | no |
| 62m | male | 1 | 1 | 0 | 0 | no |
| 63m | male | 1 | 1 | 0 | 0 | no |
| 64m | male | 1 | 1 | 0 | 0 | no |
| 65m | male | 1 | 1 | 0 | 0 | no |
| 66m | male | 1 | 1 | 1 | 1 | no |
| 67m | male | 1 | 1 | 1 | 1 | no |
| 68m | male | 1 | 1 | 1 | 1 | no |
| 69m | male | 0 | 0 | 1 | 1 | no |
| 71m | male | 0 | 0 | 1 | 1 | no |
| 72m | male | 0 | 1 | 1 | 1 | no |
| 73m | male | 0 | 1 | 1 | 1 | no |
| 74m | male | 0 | 0 | 1 | 1 | no |
| 75m | male | 1 | 0 | 1 | 1 | yes |
| 76m | male | 1 | 1 | 0 | 0 | no |
| 77m | male | 0 | 0 |  |  | yes |
| 78m | male | 0 | 0 |  |  | yes |
| 79m | male | 1 | 1 | 0 | 0 | no |
| 80m | male | 0 | 0 | 0 | 0 | yes |
| 81m | male | 0 | 1 | 1 | 1 | no |
| 84m | male | 0 | 0 | 1 | 1 | no |
| 85m | male | 0 | 0 | 1 | 1 | no |
| 86m | male | 1 | 0 | 1 |  | no |
| 88m | male | 1 | 1 | 0 | 0 | yes |
| 89m | male | 0 | 0 | 1 | 1 | no |
| 90m | male | 1 | 1 | 1 | 1 | yes |
| 91m | male | 1 | 1 | 0 | 1 | no |
| 92m | male | 1 | 1 | 1 | 1 | yes |
| 93m | male | 0 | 1 | 1 | 1 | no |
| 94m | male | 0 | 1 |  |  | no |
| 95m | male | 1 | 0 | 1 | 1 | no |
| 98m | male | 0 | 0 | 1 | 1 | no |
| 99m | male | 0 | 0 | 1 | 1 | no |
| 100m | male | 1 | 1 | 1 |  | no |
