# Supplementary material for: House sparrows do not exhibit a preference for the scent of potential partners with different MHC-I diversity and genetic distances
Source: PLoS One. 2022 Dec 21;17(12):e0278892. doi: 10.1371/journal.pone.0278892 (PMC9770374; doi:10.1371/journal.pone.0278892)
Supplement: S2 Table — (DOCX) [file pone.0278892.s002.docx]

**S2. Table.** Mean, standard error, minimum and maximum of amino acid diversity, functional diversity, amino acid distance and functional distance, as well as differences between diversity and distance.

|  | **Mean** | **SE** | **Minimum** | **Maximum** |
| --- | --- | --- | --- | --- |
| MHC amino acid diversity focal bird | 4,54 | 0,17 | 1,0 | 10,0 |
| MHC functional diversity focal bird | 4,44 | 0,16 | 1,0 | 10,0 |
| MHC amino acid diversity scent donor bird (left) | 4,97 | 0,20 | 1,0 | 10,0 |
| MHC functional diversity scent donor bird (left) | 4,74 | 0,19 | 1,0 | 10,0 |
| MHC amino acid diversity scent donor bird (right) | 4,86 | 0,20 | 1,0 | 10,0 |
| MHC functional diversity scent donor bird (left) | 4,70 | 0,19 | 1,0 | 10,0 |
| Difference in the amino acid diversity between scent donor birds | 0,11 | 0,30 | -9,0 | 9,0 |
| Difference in the functional diversity between scent donor birds | 0,04 | 0,28 | -9,0 | 9,0 |
| Amino acid distance between the left scent donor bird and the focal bird | 0,74 | 0,02 | 0,1 | 1,0 |
| Amino acid distance between the right scent donor bird and the focal bird | 0,74 | 0,02 | 0,2 | 1,0 |
| Difference in amino acid distance between the left donor and the right donor | -0,00 | 0,02 | -0,7 | 0,5 |
| Functional distance between the left scent donor bird and the focal bird | 0,71 | 0,01 | 0,3 | 0,9 |
| Functional distance between the right scent donor bird and the focal bird | 0,70 | 0,01 | 0,3 | 0,9 |
| Difference in functional distance between the left donor and the right donor | 0,01 | 0,02 | -0,4 | 0,5 |
