# Supplementary material for: House sparrows do not exhibit a preference for the scent of potential partners with different MHC-I diversity and genetic distances
Source: PLoS One. 2022 Dec 21;17(12):e0278892. doi: 10.1371/journal.pone.0278892 (PMC9770374; doi:10.1371/journal.pone.0278892)
Supplement: S3 Table — (DOCX) [file pone.0278892.s003.docx]

**S3. Table**.- Analysis of the choice of birds in relation to differences in the diversity or dissimilarity between scent donor birds. Final models for bird choice (left vs right side of the olfactometry chamber) in relation to sex. difference in body weight and the difference in a) MHC amino acid diversity, b) functional diversity, c) Amino acid distance, d) Functional distance between the scent donor bird of the opposite sex that was providing the scent in the left side of the chamber and the one that was scent-donor in the right side of the chamber, controlling for the cent donor pair as a random factor and for knocking and side as a fixed factor.

1. Amino acid diversity

|  | Estimate | SE | *Z* | *P* |
| --- | --- | --- | --- | --- |
| Intercept | 0.01 | 0.48 | 0.03 | 0.98 |
| Difference in body weight | 0.06 | 3.48 | 0.02 | 0.99 |
| Difference in amino acid diversity | 0.08 | 0.12 | 0.67 | 0.50 |
| Sex | -0.80 | 0.46 | -1.75 | 0.08 |
| knocking | -0.003 | 0.24 | -0.01 | 0.99 |
| Side | -0.30 | 0.85 | -0.35 | 0.73 |

1. Functional diversity

|  | Estimate | SE | *Z* | *P* |
| --- | --- | --- | --- | --- |
| Intercept | -0.03 | 0.47 | -0.06 | 0.95 |
| Difference in body weight | -0.04 | 3.67 | -0.01 | 0.99 |
| Difference in functional diversity | 0.06 | 0.13 | 0.50 | 0.61 |
| Sex | -0.77 | 0.45 | -1.69 | 0.09 |
| knocking | -0.05 | 0.24 | -0.19 | 0.85 |
| Side | 0.03 | 0.86 | 0.04 | 0.97 |

1. Amino Acid distance

|  | Estimate | SE | *Z* | *P* |
| --- | --- | --- | --- | --- |
| Intercept | -0.01 | 0.39 | -0.03 | 0.97 |
| Difference in body weight | -0.51 | 3.25 | -0.16 | 0.87 |
| Difference in amino acid distance | -0.26 | 1.22 | -0.22 | 0.83 |
| Sex | -0.89 | 0.42 | -2.13 | 0.03 |
| knocking | 0.12 | 0.22 | 0.55 | 0.58 |
| Side | -0.27 | 0.60 | -0.45 | 0.65 |

1. Functional distance

|  | Estimate | SE | *Z* | *P* |
| --- | --- | --- | --- | --- |
| Intercept | -0.13 | 0.41 | -0.32 | 0.75 |
| Difference in body weight | -0.47 | 3.21 | -0.15 | 0.88 |
| Difference in functional distance | 0.29 | 1.50 | 0.19 | 0.85 |
| Sex | -0.89 | 0.41 | -2.17 | 0.03 |
| knocking | 0.15 | 0.22 | 0.69 | 0.49 |
| Side | -0.07 | 0.58 | -0.12 | 0.91 |
