# Supplementary material for: House sparrows do not exhibit a preference for the scent of potential partners with different MHC-I diversity and genetic distances
Source: PLoS One. 2022 Dec 21;17(12):e0278892. doi: 10.1371/journal.pone.0278892 (PMC9770374; doi:10.1371/journal.pone.0278892)
Supplement: Attachment — Submitted filename: Review for PONE-D-22-00952.pdf [file pone.0278892.s006.pdf]

This study adds to the expanding field of avian chemical communication by studying the olfactory preferences of house sparrows as they relate to MHC-I genes. The authors find no preferences in either males or females. I think this is first study to look at MHC-I genes and olfactory preferences in birds, so this manuscript represents an important contribution to this growing field. I thought the writing could be benefit from some re-organization and editing to improve clarity and increase readability as I found it difficult to follow in certain places. I have tried to provide extensive comments to help identify the places where I think some changes would be beneficial. I also have several concerns relating to the decision to analyze continuous genetic distance/dissimilarity variables as a dichotomous categorical variable, and some questions about interpretation of the results, particularly the new supplementary results that were added in the prevision round of revisions. I have divided by review up with comments or questions for the various sections of the manuscript.

Introduction

I think the introduction could be more streamlined and the organization could be improved to better convey the important points. I have provided multiple suggestions below.

Paragraph 1:

This paragraph starts out discussing female choice and then makes a diversion to male choice in red jungle fowl in the last sentence. This currently reads awkwardly. Since the experiment described in this manuscript tested the ability of both males and females to discriminate MHC dissimilarity/diversity in opposite sex individuals, I think this entire paragraph would be stronger if it discussed mate choice as a process where both males and females may participate. This is a reasonable assumption for house sparrows (and many bird species) where there is some degree of biparental care. While females might be more motivated to be choosey because they invest more in reproduction, males are also contributing to parental care and therefore may also participate in mate choice. I assume the authors agree otherwise they would not have bothered to test the olfactory preferences of males. Lastly, a study in birds that documented MHC-based mate choice by males (for MHC Class II) is Hoover et al. 2018 Molecular Ecology (https://onlinelibrary.wiley.com/doi/abs/10.1111/mec.14801). This could accompany the red jungle fowl citation to help strengthen the discussion of MHC-based mate choice by males.

Paragraphs 2 and 3:

MHC is not explained until Line 78, halfway through paragraph 3, but there are multiple references to MHC in the second paragraph (and also at the end of the first paragraph). This is not a great approach because it assumes the reader is already familiar with MHC. I recommend that the information from Lines 78 through 84 is presented a lot earlier in the introduction to avoid confusion.

The text in these paragraphs repeatedly uses the terms “genetic dissimilarity” and “heterozygosity” but it is unclear if this refers specifically to MHC genes or more widely to genome-wide heterozygosity or genetic dissimilarity at other loci. There are several studies from birds that have documented covariation between non-MHC genetic markers and odor profiles (see Leclaire et al. 2012 Proc B https://royalsocietypublishing.org/doi/10.1098/rspb.2011.1611 and Whittaker et al. 2019 Animal Behaviour

https://www.sciencedirect.com/science/article/abs/pii/S0003347219300831). It should be clarified whether the studies included here are specifically relating to MHC genes or not.

Paragraph 4:
Line 102. It is unclear to me why the findings of Leclaire et al. and Grieves at al. are being described as "controversial". Is this word used because many people assume birds are unable to use odors in social contexts? I think this choice of word could come across as negative towards these past studies and I do not believe that is not the intention of the authors. I recommend the wording is changed here.

In my opinion, there are three pieces of evidence that work together to support olfactory-mediated MHC-based mate choice in birds (or any vertebrate species):

1. Observations of non-random mating associated with MHC genes. There are a decent number of published studies in birds.
2. Studies showing that chemical profiles that contain information about MHC genes. In birds, this has been shown for preen oil (Leclaire et al. 2104 Scientific Reports https://www.nature.com/articles/srep06920, Slade et al. 2016 Proc B https://royalsocietypublishing.org/doi/10.1098/rspb.2016.1966#sec-7) and plumage (Jennings et al. 2022 Proc B https://royalsocietypublishing.org/doi/full/10.1098/rspb.2022.0567).
3. Behavioral experiments where individuals exhibit olfactory preferences for individuals based on their MHC genes.

This study clearly falls into the third category, but all three pieces are needed to make a strong case for MHC-based mate choice via olfaction. The introduction of this manuscript has several citations for MHC-based mate choice and there is a discussion of the behavioral evidence in the 4th paragraph. I think it would strengthen the introduction to briefly touch on the evidence from chemical profiles. This may help to convince anyone skeptical about the avian sense of smell that this area of research deserves more attention. I also think this is relevant to the non-significant results obtained in this study. One of the outstanding questions that remains based on the findings of this manuscript is whether or not house sparrow body odor contains information about MHC-I. One possibility is that their body odor does not reflect information about MHC-I. Alternatively, information about MHC-I may be present, but the birds either do not detect it via olfaction or do not use it in the context of mate choice. These points could be easily added to the discussion if this idea had already been introduced in the introduction.

Methods

I had a number of questions about the details of the preference tests. I have grouped all of my comments together.

Timing of each trial:
I found the protocol for the behavioral trials difficult to follow. I believe there was a 5-minute acclimation period during which the focal bird was exposed to the odors from the two donor birds. After 5 minutes, the two trap doors were opened by the human observer and the focal bird was given one minute to make a choice. If they did not make a choice after 1 minute, the human observer tapped on the arena and the side the bird moved to following the tap was noted as their choice. The total duration of the experiment was 6 minutes per trial. I think this is correct, but it

took me reading the methods section several times to get all this information. I struggled to understand the protocol because the section about the “Behavioural Experiment” jumps around a lot. The reader learns about the 5 min acclimation period in Line 205, but the 1 min period during which the bird is allowed to make a choice is not discussed until Line 228, almost an entire paragraph later. It is easy for the reader to lose track of what is going on. Interspersed between these two protocol details is a lot of information about the design of the experimental arena. I recommend that this section is restructured so that the design of the arena with its various compartments, fans, how the focal bird received the odors etc is provided first. Then, move into the specifics of the protocol for the experiments with the different timings (5 mins, 1 min, tap on arena etc).

Secondly, it reads to me like all individuals in the study made a choice. That is often not the case for olfactory preference tests where many protocols remove the individual after a certain amount of time and mark the trial as a “no choice” result. If would be helpful if the methods stated that the tapping always resulted in the bird selecting a donor bird. Somewhere around Line 231 seems like a logical place for this information.

Size of cages:
Please add the dimensions of the two little cages discussed on Line 207 where the donor birds were housed.

Visual cues in the experiment:
I believe all the birds (donor and focal) were in the dark to eliminate visual information. Line 205 says “the bird was maintained in the dark for 5 min” and Line 216 says “donor birds were kept in darkness for the entire trial duration. Lastly, on Line 222 it says, “the chamber was opaque to maintain birds in darkness”. This all seems fine except I am unsure how the human observer who scored each trial was able to see which donor bird the focal bird selected under these conditions. Was the light in the center chamber turned on after the 5 min acclimation period? How exactly did this work? Was the observer able to hear where the bird moved and did not need to make a visual observation? Please add details to the methods to clarify.

Pairing of donor birds based on morphometrics:
The two focal birds used in the trials were matched for a number of visual characteristics including body size, weight, and size of badge. As the focal bird was unable to see the donor birds, I assume this was done to remove any potential correlation between body condition/dominance status and avian body odor? I believe the intention was to have MHC be the primary factor that differed between the two donor birds so that any differences in odor profiles could most likely be attributed to MHC and not another confounding factor. If this is correct, I think it would be beneficial to clarify this in the paragraph that begins on Line 233. Otherwise, it is not obvious why so much care was taken to match visual traits for the donor birds in an experiment that only examined olfactory preferences.

Data Analysis

I have a number of questions relating to the data analysis. If I had to replicate this analysis, I would have a hard time doing so because I am not entirely sure how each of the models are actually different. In certain places I have specific questions or comments. Throughout my comments, I have done by best to explain what I think was done. I hope this will help the authors

make edits to clarify their methods, particularly in places where I have misunderstood the analysis.

I think there are two ways to examine the behavioral data. Each trial has a triad of individuals; one focal bird who performed the experiment and two donor birds that were used as scent sources. One set of analyses could compare the MHC genotypes of two donor birds to each other (ignoring the genotype of the focal bird). This could be done by measuring the number of amino acids encoded by each donor genotype or by obtaining a measure of functional diversity for each donor genotype. This type of analysis would determine if there was a preference by the focal bird for individuals with higher MHC diversity. A second set of analyses could examine the MHC genotype of the donors relative to the MHC of the focal bird. This approach differs from the first because it considers the genotype of the focal bird, which is important as individuals may be looking for a mate with a genotype that complements their own rather than simply preferring a mate who has maximum genetic diversity (a complementary mate vs a genetically diverse mate). This analysis would require measuring the genetic distance between focal and donor birds, which could be based on amino acid differences or functional differences. This analysis would test whether the focal bird more often selects an individual who has more dissimilar MHC relative to their own genotype. I believe both these approaches were implemented to some degree in this study, although I am not entirely unsure.

There are four main analyses described. I have done my best to describe what I think is going on in each analysis.

The first analysis is described in the paragraph starting on Line 261. It used amino acid variant number. Based on the information given in Line 266, it reads as if the number of amino acid variants of each donor bird was compared to the number of amino acid variants in the focal bird, and the two donors were classified as having either higher or lower numbers of amino acids than the focal bird. The model tested the probability that the focal bird preferred the donor with the higher number of amino acid variants than the donor with the lower number of amino acid variants. This type of analysis is similar to the second option I described above because it considers the genotype of the focal bird. I have two comments related to this analysis:

- Please give a definition or description for what “amino acid variant numbers” means. Is this the number of amino acids encoded by their various MHC alleles?
- As the MHC of the focal and donors was unknown during the experiment, how many trials occurred where both of the donor birds had a higher amino acid variant number compared to the focal bird? Similarly, both of the donor birds had a lower amino acid variant number compared to the focal bird? Another way of putting this is, how many trials did you have to exclude from your analyses after you obtained the genotype information?

A second analysis is described in the paragraph starting on Line 275. It looks at the number of MHC functional alleles. This model includes the number of functional MHC alleles for the two donor birds and the number for the focal bird (I assume as a covariate), but it does not measure the difference in functional alleles between the focal bird and each of the donor birds. I think this is similar to the first type of analysis I described above.

Lastly, two other analyses are described in the paragraph on Line 282. Both seem to be similar to the second type of analysis I have described above because they consider the difference in MHC between focal and donor birds. The first measures this difference using MHC-I amino acid distance and classifies each donor as having higher or lower. For example, if the amino acid distance between focal and donor 1 was 4 and the distance between focal and donor 2 was 18, the focal-donor 2 pairing would be assigned "higher" and focal-donor 1 pairing would be assigned "lower", I think? Similarly, a second analysis was done in the same way except this time the genetic distance was measured as functional distance. If this is not correct, please consider making edits to clarify. Also, I have several specific questions:

- I am unclear how amino acid distance is different from the amino acid variants that was used in the first analysis. Possibly a better description of amino acid variants as I requested above would help clarify this.
- Line 287: "in relation to the sex of the focal bird". Why is the term "sex" used here?

Line 290: please state the version of R that was used (e.g., 4.2.1) and double check the citation information. The current citation says 2012, which seems incorrect as I suspect the authors used a more recent version of the software for their analyses.

I have a general question that relates to the analyses in the main text. Why were the data turned into a dichotomous variable of lower or higher? It seems that for each of the genetic distance/dissimilarity measures, there should be actual numbers (aka continuous variables) that describe the magnitude of the difference between different individuals. I think the analysis would be far more powerful if the values were kept. By converting to higher/lower you are treating trials as similar that could have been quite different. Here is an example:
Trial 1: distance between focal-donor 1 is 2, distance between focal-donor 2 is 3. In this example, focal-donor 1 would be classified as lower and focal-donor 2 as higher. In reality though, there is very little difference between the pairings, and we may expect that the focal bird would not have very strong preferences in this situation.
Trial 2: distance between focal-donor 1 is 15, distance between focal-donor 2 is 3. In this example focal-donor 1 would be classified as higher and focal-donor 2 as lower. Now there is a very large difference between focal-donor 1 and very little difference between focal-donor 2. If house sparrows are evaluating MHC-I using odors, I expect this scenario could play out differently than above. I think the authors need to better justify their decision to use the dichotomous classification as it is unclear to me why this was done, and it seems to result in a loss of information that make their results less conclusive.

Results

I have two main concerns relating to the results and the current interpretation of these findings.

On Line 245-248 the manuscript says "Focal birds made 61% of their first choices to the left and 39% of the choices to the right side. There were not significant difference in the number of scent donor birds more diverse or more dissimilar between those located in the right side or left side of the aviary". This seems somewhat concerning. If there was no trend in the placement of more diverse or more dissimilar donors, then a strong preference for the left side of the aviary suggests that the choice made by the focal bird was less about the odor presented and possibly associated

with a spatial preference for the left side that was unrelated to the presented odors. The models described in the data analysis do not appear to account for whether the choice was on the left or right side. I think this result needs to be carefully considered.

On Line 382 some preliminary analyses are mentioned. The findings from these analyses are given in supplementary Table 1. I think these analyses are similar to the analyses in the main text, but they used the genetic distance measures as continuous variables, which I think is actually a more powerful approach (as I have discussed above). This section goes on to conclude "therefore, we can exclude that the differences in MHC-I diversity were very small and house sparrows were not able to detect them" (Line 387). I do not agree with this statement based on the findings presented in supplementary Table 1. In the lower half of this table, the differences between the two focal birds, or the differences between each focal-donor pairing are given. The mean distance for each of these is very low and often very close to zero. My interpretation of these values is that for most of the trials there was very little difference in MHC-1 between all 3 individuals used. If this was the case, this could be a very important reason why there were no significant findings in this study, which is the opposite of what is stated in Line 387. We would not expect the birds to exhibit strong olfactory preferences if the two donor birds had very similar MHC and/or the two donors had similar MHC relative to the focal bird. These values either need to be explained more clearly or the authors need to reconsider their interpretation of these values. I also have two minor comments relating to Table 1:

- Use decimal places rather than commas for the values in the table.
- In the middle of the table, two rows have minimum values of -9 and positive values of +9. Is this a type or is this correct? It seems odd that these values would be the same for both amino acid diversity and functional diversity.

Discussion

The discussion focuses extensively on the methods of the experiment and how the methods may explain the results. This is fine but I think the paper would be stronger if it devoted a little more space to biological explanations and interpretations of the findings. The paragraph starting on Line 400 is the most interesting and I have provided some additional ideas to potentially expand the discussion.

Line 374: this is a pretty extreme statement. I recommend toning down the language a little. Notably, the wording "could hardly be explained" is rather strong. I think it is fair to say that house sparrows use olfaction in other social contexts, so it seems probable that odors also play a role in mate choice. However, there is still a lot that is unknown about the sense of smell in this species and birds in general.

Secondly, assuming house sparrows do have a great sense of smell and use olfaction to evaluate mates, there seems to be two possible explanations that could be driving the lack of significant findings. First, as I discussed above, the body odor of house sparrows may contain no information about MHC-I genes. I think this should be mentioned in the discussion

Moreover, another potential factor driving the results of this manuscript is the influence of other MHC genes or other genetic markers on the odor of the birds, and thus their preferences in this experiment. Most of the evidence in birds that connects chemical profiles, olfaction, and social

interactions has focused on MHC-II. This has been the focus because of its connection to the microbiome and the ability of bacteria produce volatile chemicals. As is pointed out in this manuscript, birds lack a vomeronasal organ and therefore likely only sense volatile chemicals using their olfactory system. Volatile information about MHC-II may be more important to house sparrows and driving their choices in this experiment. Furthermore, non-MHC genes can shape chemical profiles, and birds could be using odors associated with other genetic markers to form preferences. I would like to see more discussion around how other genes that were not examined in this study could be driving the results.

One aspect of this study that I thought was particularly strong was the use of live birds as the scent source. This is in contrast to the Leclaire et al. 2017 which used cotton swabs and bags as the odor source and Grieves et al. which used a sample of preen oil. While preen oil is thought to be the primary source of chemicals that make up avian body odor, there are differences between the compounds on bird feathers and the preen oil (see Mardon et al. 2011 J Avian Biology https://onlinelibrary.wiley.com/doi/abs/10.1111/j.1600-048X.2010.05113.x)
Arguably, using a live bird is the most accurate approach because it ensures all the odorants are present, even if the sounds and movements produced by live birds may complicate things. The authors may want to consider including slightly more discussion on their decision to use live birds and why this is a strength of their study.
