# Supplementary material for: House sparrows do not exhibit a preference for the scent of potential partners with different MHC-I diversity and genetic distances
Source: PLoS One. 2022 Dec 21;17(12):e0278892. doi: 10.1371/journal.pone.0278892 (PMC9770374; doi:10.1371/journal.pone.0278892)
Supplement: Attachment — Submitted filename: PONE-D-22-00952_ReviewerComments.docx [file pone.0278892.s004.docx]

This paper tests for evidence of odour-based discrimination in birds based on MHC class I diversity and similarity. As an understudied area of research, this paper makes a useful contribution to the literature, but I have two main concerns I would like to see addressed before recommending for acceptance: presentation of methods and data, and the focus and scope of the discussion. I discuss in more detail below.

**Presentation of methods and data:**

It is essential to present at least a summary (e.g. mean +/- SE) of the MHC class I diversity and distances of the stimuli presented to the focal birds. Such data are necessary to evaluate the differences in allelic diversity and genetic similarity of the paired stimuli birds were tested with. For example, it is possible that birds did not respond differentially to donor bird odours because differences in MHC diversity +/or similarity were very small – if that may be the case, it is important to discuss whether a detection capability threshold based on MHC diversity/similarity was likely to have been met or not.

Relatedly, if the distances and diversity differences were very small, I recommend the authors analyze the subset of maximally different stimuli pairings (using some reasonable threshold determined a priori) to see if the focal birds presented with maximally different pairings responded differently.

Given that these were post hoc tests, it seems that these behavioural trials were not originally designed to test MHC-based odour preferences. The authors should be clear about this and state how and when these data were originally used. I don’t necessarily think it’s inappropriate to evaluate the data post hoc, but I think it is important to be clear and up front about this, and to clearly reference any other papers in which these data have been previously used.

**Focus and scope of discussion:**

Related to the methods and study design, the authors should discuss limitations of the study. For example, with respect to whether the MHC diversity and similarity distances were likely appropriate/sufficient for detection by house sparrows.

Currently, the authors spend a lot of time discussing the validity of using first choice, and of tapping on the maze after 1 min to force a choice, but they do not discuss the possibility that this method may not be as appropriate for passerines or sparrows in particular, compared to other species in which first choice is more commonly used, such as seabirds, which may reasonably be expected to behave differently in the test chamber than sparrows do. The authors argue that many studies use this method, but they do not point out that at least as many studies (if not more) use cumulative time spent with a sample type, also with good success (especially for passerines).

Finally, there is no discussion of how the timing of the experiment and the physiological condition of the birds could have impacted results or predictions of focal bird preference. In the Leclaire study referenced, birds were incubating and may have preferences related to this, whereas in the Grieves study birds were in breeding condition and may have been motivated differently than in the Leclaire study. This should be included in the discussion alongside consideration of the breeding condition of the birds used in the author’s present study and how this may have affected results and/or predictions/expected outcomes.

**Detailed comments:**

Line 86: why would MHC I be related to bacterial loads?

Lines 93-101: odd to name authors of one study but not the other, and name the study species in one study but not the other. Suggest rephrasing both sentences for consistency of results presentation. It is also worth explaining here or in the discussion the different predictions that can reasonably be made for each sex at different breeding stages (such as during mate choice and pairing vs during incubation) or different predictions that might be made for different taxonomic groups (seabirds vs songbirds) that may differ in level of parental care and investment between the sexes (sex roles, timing of care/investment for each sex, etc).

Line 101: results of Leclaire and Grieves are conflicting but are they controversial? Perhaps not, especially when considering time in breeding cycle as described above.

Line 115: MHC I, II, or both?

Line 120: high or intermediate MHC diversity

Line 133: when was the genotyping performed? Birds were captured Feb-Mar 2015 but it’s not clear when genotyping was done in relation to when the behavioural trials were conducted.

Line 136: given that all birds were initially allowed to interact in a group aviary, could it be that mate choice had already taken place, or preferences have otherwise been established? Should discuss whether/how this may have impacted results. Was any evidence of courtship or breeding activity observed during the time birds were group housed?

Line 143: include primer references/original source for primer development

Line 144: please describe what is meant by 'illumina sequencing primer sequences' in more detail so methods can be evaluated.

Line 148: please explain how index sequences differ from illumina sequencing primer sequences and why these were attached in a separate round of PCR

Line 149: demultiplexing?

Line 158: what criteria were used to set this 1% threshold? Overall, more information on sequence data processing methods is needed to properly evaluate these methods. The only reference given is 10 years old and, given the rapidly changing nature of data handling methods in this field, it is essential to ensure the most up to date and appropriate methods are being used for this type of data.

Line 159-160: rarefaction is not appropriate for these data (see McMurdie, P.J. and Holmes, S., 2014. Waste not, want not: why rarefying microbiome data is inadmissible. *PLoS computational biology*, *10*(4), p.e1003531.).

In Strandh et al. 2012, this 200 cutoff was based on inclusion of technical duplicates, which his reliable only within the sequencing run being evaluated. Were technical duplicates included in this study? If so, this procedure must be described and explained. If not, this method is inappropriate and should not be used. Instead, please update with more recent/current methods.

Line 169-170: JTT SMS explain/write out in full on first mention

Line 201: was the light turned on or was it kept dark throughout the trial?

Line 217: was experimenter present the entire time? Was experimenter visible to the focal / test birds? Was the test chamber opaque or transparent?

Line 224: admittedly, I struggle to accept this method as being appropriate... Regardless, it is important to present a summary of how many birds was this done for. A 1 min trial duration seems very short. Many similar studies allow 15 min for birds to choose, even those scoring first choice. I recognize you cannot change this now, but in future, why not use a longer trial duration and evaluate both first choice and choice / time?

Line 232: why were only 120 of the original 151 birds tested?

Line 235: please include discussion of the side bias exhibited by test birds. How often was the more vs less diverse and more vs less similar individual placed in the left or right side of the olfactometry chamber?

Line 237: what type and % of alcohol was used?

Line 266: what were the distances? How was it determined that this was a detectable threshold or that differences in allelic diversity and genetic similarity were otherwise reasonable? Must present data on what MHC distance and allelic diversity pairings were used.

Line 286: these trials appear to have been used for multiple experiments/to test multiple questions post hoc. If so, this should be clearly stated somewhere in the methods.

Line 316-321: again it's odd presentation to name author in one study and not the other, suggest rephrase so both are consistent.

Line 321: song sparrows are relatively asocial, blue petrels relatively social, and house sparrows relatively social, but all species may differ in level of sociality during breeding – please discuss how you think sociality may be involved.

Line 326: many of these studies set a maximum trial duration of 15 min, presumably to allow time for the birds to choose on their own. The present study seems to allow the birds only 1 min, followed by tapping on the test chamber to force the birds to move, which while use in prior studies of this group. The potential impacts of this should be discussed.

Line 327: but what MHC differences were used in terms of both diversity and dissimilarity? Again, this information needs to be provided.

Line 331-341: this would be a good place to discuss the results in terms of the relative differences in MHC I diversity and similarity of the stimuli, and how this may have affected the results.

Line 342: returning back to a justification of the first choice method is jarring here; what does this have to do with future work or using preen oil instead of whole body odour? Why would preen oil be superior to use of whole body odour? Suggest restructuring the relevant paragraphs for clarity/flow.

Line 347: potential mechanisms for scent-cues of MHC genotype would be better placed in the introduction to set up the study rationale. Since the study did not find evidence for odour choice, I don’t think this discussion of potential mechanisms needs to be here.

Line 353: birds lack a VNO – what is the relevance of including this information here?

Line 354: would MHC I be expected to be involved in influencing bacterial profiles?

Lines 357-360: please put this information in the context of the present study.

Line 374: a lot of time is spent early in the discussion justifying the use of first choice, but now seems to conclude by saying that different methods should be used - what methods should be used/are recommended? Isn't it worth suggesting that choice over time (cumulative time spent with stimulus) might be a good approach to consider for this type of research in songbirds? First choice has been used successfully in several studies, but so has cumulative time spent, and this should be acknowledged and evaluated. Overall, I think the discussion should be reorganized and more tightly focused around the research question and study results.
