# Supplementary material for: House sparrows do not exhibit a preference for the scent of potential partners with different MHC-I diversity and genetic distances
Source: PLoS One. 2022 Dec 21;17(12):e0278892. doi: 10.1371/journal.pone.0278892 (PMC9770374; doi:10.1371/journal.pone.0278892)
Supplement: Attachment — Submitted filename: Response to Reviewers.docx [file pone.0278892.s005.docx]

**Response of comments of Editor:**

Additional Editor Comments (if provided):

The manuscript is well written and easy to follow, and the experimental design as well as the results analyses are correct.
Maybe it is not very clear in the variables such as "amino acid variants", "amino acid distances" and "functional alleles", the mening of "more" and "less", when it is considered in each category.

Response: Thank you for the overall positive evaluation of our ms. We have tried to clarify and be more concise in the definition of each category. We have provided a supplementary material file adding the mean ± SE of those variables as well as a statistical analysis of differences in these variables between the scent donor partners in relation to the choice of birds.

**Response to comment of Reviewer 1**

Review Comments to the Author

Reviewer #1: The study investigates whether assortative mating for MHC-I diversity and dissimilarity is mediated by olfactory cues in a passerine bird, the house sparrow. Specifically, the authors experimentally test through behavioural observations if males and females show a preference for potential mating partners that differ in MHC-I amino acid and functional variance as well as in their similarity. Neither males nor females showed a significant preference for conspecifics differing in MHC-I amino acid and functional diversity or dissimilarity. Avian olfaction has recently gained interest in ornithology and behavioural ecology. However, the implications of olfactory perception in bird biology and evolution are for the most part still obscure. This study tries to fill the knowledge gap. The experimental hypothesis builds on a solid amount of literature and I’m glad that it has finally been tested, especially in this bird species. The manuscript is well-written, with sound statistical methods, and the conclusions follow the results obtained. However, some sections should be further improved before acceptance, in particular the discussion (see below).

Response: Thanks, we have tried to improve the ms following your suggestions.

Major comments:
1. ll. 53-56: Not clearly written. I guess here you want to say that the increased use of molecular techniques for assessing paternity and genetic compatibility showed that females can choose mates also based on traits and/or mechanisms other than the “good genes” hypothesis? Please, clarify. Also, keep in mind that mate choice can also occur in males. Since this study tested also for male mate choice I would definitely not narrow the reasoning to female mate choice in this entire paragraph. Finally, I would like to see a (review) reference to the different mechanisms of assortative mating to which you are referring to.

Response: Thanks, we have tried to improve this paragraph (lines 59-60): “In contrast, studies addressing the mate choice of females in relation to genetic dissimilarity are less well described. However, the increased use of molecular techniques for assessing paternity and genetic compatibility showed that females can choose mates also based on traits and/or mechanisms other than the “good genes” hypothesis, but indeed suggest additional mechanism critical to female choosiness. Females may thus use alternative mechanisms for choosing mates such as extra-pair copulations [6, 7], or post-copulatory choice [8]. Furthermore, molecular techniques have also revealed that male choice may also use alternative mechanisms for selecting females (Gillingham *et al*. 2009). For example, red jungle fowl (*Gallus gallus*) males decrease their investment in reproduction, i.e., by allocating less sperm, when copulating with females with similar MHC alleles (Gillingham *et al*. 2009).”

2. ll. 56-57. Extra-pair copulations and post-copulatory choice are specific mechanisms through which females modify the paternity of the offspring rather than mechanisms of choice of (social) mates and I would be careful in mixing the two. Please, explain better or remove the sentence.

Response: Our aim was to describe mate selection, not only social partner selection, so, we have removed the sentence: “In fact, in many passerine species, male parental care of offspring is needed to ensure nestling survival.”

3. ll. 65-67. From this sentence it is unclear why females should do that only when engaging in extra-pair copulations. Please, add details or remove “when engaging in extrapair copulations”.

Response: Thanks, we have tried to explain this better “For example, although blue tit *Cyanistes caeruleus* females are known to use song [12] and plumage coloration [13] when selecting social mates, there is evidence that they select extra-pair partners based on their genetic dissimilarity, as extra-pair nestlings showed higher degree of heterozygosity compared to within-pair nestlings [14]. Since nestlings with higher degree of heterozygosity were more likely to survive [14], females may increase their fitness by selecting genetically dissimilar mates when engaging in extra-pair copulations. Although, females may ensure all nestling survival by choosing the best parental male based on phenotypical characteristics such as plumage coloration [13], they may indeed use other characteristics signalling male genetic heterozygosity [15, 16].

4. ll. 124-126. As far as I understand the badge size was used to account for male dominance status (l. 229). I’m not against doing this but the reliability of the badge size as signal of dominance in house sparrows has recently been questioned and the reader should be warned about this. See: Sanchez-Tojar, Alfredo, et al. "Meta-analysis challenges a textbook example of status signalling and demonstrates publication bias." Elife 7 (2018): e37385, <https://doi.org/10.7554/eLife.37385>.

Response: Thanks, we have added this reference.

5. l. 201. How were doors opened? Was it remotely or the experimenter physically got close to the bird and opened them manually? Add details to understand if the focal bird may have been startled during the opening phase.

Response: We have added that “the doors were remotely opened with a rope”. Therefore, the chamber was not directly approached by the experimenter and did not scare the birds while opining the doors.

6. ll. 221-224. How long did every test lasted?

Response: We have explained that “Tests lasted up to 6 minutes”.

7. l. 226. How did you calculate body condition? Was it just body weight?

Response: Yes, we have changed body condition to body weight.

8. ll. 234-235. Out of the 68 pairs, how many were males and how many females? Only pairs of the opposite sex respect to the focal bird were tested, correct? State it clearly

Response: We have added that we used: “41 pairs of females and 27 pairs of males as scent donours”. We already said that we offered focal birds the scent of two potential mates of similar body condition and dominance status in the case of males. So, it is stated.

9. ll. 319-321. Before discussing the methodological differences, there are two (quite obvious) hypotheses that are not being mentioned and that I recommend discussing. First, passerine birds are among the bird species with the lowest olfactory bulb size (maybe this could also be mentioned in the introduction?). Hence, a reduced olfactory performance compared to other species, in particular versus procellariiformes that are on the opposite side of the spectrum, can be expected. See for instance: Corfield, Jeremy R., et al. "Diversity in olfactory bulb size in birds reflects allometry, ecology, and phylogeny." Frontiers in neuroanatomy 9 (2015): 102, <https://doi.org/10.3389/fnana.2015.00102>. Second, there is no mention to the hypothesis that house sparrows may perceive the odour cues and mate assortatively based on the MHC-II alleles (not investigated here) rather than based on the MHC-I alleles. This is particularly relevant as some studies that you also mention (references nr. 7, 9, 15, 16 and 66) and that are central in supporting the hypothesis on the significant role of MHC in avian mate choice, focused on MHC-II alleles instead of MHC-I alleles.

Response: In relation to the first hypothesis you mention, there is evidence that house sparrows can use olfaction in social contexts. We have mentioned this in the discussion: “The absence of odour-based preference for more diverse or more dissimilar potential partners could hardly be explained by a lack of olfactory abilities in house sparrows because house sparrows are known to use olfaction in social contexts [Fracasso et al. 2019], so a low olfactory capability is also not likely to explain this lack of preference.” In relation to the second hypothesis you suggest, it is true that house sparrows may perceive the odour cues related to MHC-II alleles instead of MHC-I alleles, but previous studies have shown that they can mate assortatively based on the MHC-I alleles [42], so we think it is worth to examine whether they may also assess the MHC-I allele characteristics of potential partners. Ultimately, we have mentioned that maybe house sparrows use olfaction to assess MHC-II status of potential partners: “However, while there is no reason to expect that all bird species use the same type of information to evaluate potential partners, our results are difficult to explain in a sexual context, because in house sparrows MHC-I diversity is associated with reproductive success [40]. It is also known that males with low degree of MHC-I diversity or too dissimilar MHC-I alleles fail to form breeding pairs [41]. Furthermore, results of a previous study showed that house sparrow females with low MHC-I allelic diversity choose males with high MHC-I allelic diversity [42], suggesting that house sparrow females show mate preferences based on the MHC-I of potential partners ([42], but see [43]). Collectively, in this study we tested whether olfaction is mediating olfactory recognition of MHC-I, but further experiments are needed to test whether house sparrows may use olfaction to assess MHC-II similarity of potential partners, as has been shown in blue petrels [15] and song sparrows [16].”

10. l. 346. Could the use of olfactory cues for mate choice be seasonal?

Response: Yes, the response to olfactory cues of conspecifics may be seasonal, but we performed the experiment during the breeding period with birds that were not reproducing, as they were separated by sex. Therefore, we may expect they were looking for a partner. We have explained in the discussion: “Differences between our results with house sparrows and those with blue petrels can be due to differences in the breeding conditions of birds between the studies, because Leclaire and collaborators tested blue petrels that were incubating [15] and thus, not searching for a partner. In contrast, we performed the experiment during the breeding period with birds that were separated by sex, and therefore, they were not paired or incubating, so, they may be interested in searching for a partner. Differences between our results and those obtained with song sparrows cannot be attributed to differences in the breeding condition of birds, as in both studies birds were tested during the breeding period and were not incubating. Several explanations may explain the lack of preference for MHC-I found in our study system. Previous experience with scent donour birds may have masked a preference for MHC-I diverse or dissimilar partners. However, we captured birds during February and March and kept females separated from males. Birds were separated by sex in different aviaries, to avoid establishing any interaction with potential partners that may have affected olfactory preferences. Therefore, neither the timing of the experiment nor previous interactions during the breeding period or the physiological condition of the birds explain the lack of preference for MHC-I dissimilar potential partners.”

11. ll. 372-374. This information has already been mentioned earlier in the study and it does not lead to further discussion here. I suggest to remove it.

Response: We have removed the sentence.

12. l. 634. Try to merge together table 1 and 3 as well as tables 2 and 4 if possible. The legend of each table can be reduced since all the information is also in the methods section.

Response: Thanks, we have merged table 1 and 3 in table 1a. and 1.b, and table 2 and 4 in table 2.a. and 2.b. and we have reduced the legend.

Minor comments:

13. l. 26. …selecting males with genetically compatible or heterozygous MHC.

Response: Thanks, we have changed the sentence.

14. l. 27. evidence suggests.

Response: Thanks

15. l. 31. number of MHC.

Response: Thanks

16. ll.32-35. The sentence is long and hard to read. Please, split it in two parts.

Response: thanks, we have split it.

17. l. 36. responsible for mate discrimination based on MHC-I in birds.

Response: Thanks.

18. ll. 45-50. Too long sentence. Please, split it in two parts. For instance “…[5]. These traits are…”.

Response: Thanks, we have split it.

19. ll. 50-51. The sentence doesn’t really follow the previous one. Move it after the first sentence of the introduction or remove it.

Response: We have removed it.

20. l. 58. Remove “alternative”. Alternative sexual selection doesn’t really exist.

Response: We have removed it.

21. l. 60. extra-pair relationships can. Please write “extra-pair” consistently throughout the text.

Response: Thanks, we have written extra-pair throughout the text.

22. l. 63. Remove “in”.

Response: Thanks.

23. l. 70. characteristics signaling male genetic heterozygosity.

Response: Thanks.

24. l. 80. Write MHC-I (or MHC class I) and MHC-II consistently throughout the text.

Response: Thanks, we have written MHC-I throughout the text.

25. l. 90. Well-described.

Response: Thanks.

26. l. 93. Parenthesis opens but doesn’t close.

Response: Thanks, we have corrected this.

27. l. 99. During the breeding season.

Response: We have written during the mating period, as we concretely referred to this period because the incubation period is also included in the breeding season.

28. l. 100. Females and males.

Response: Thanks.

29. l. 133. 100% ethanol? Add the percentage.

Response: We have added 94% ethanol.

30. l. 151. Remove space before %.

Response: Thanks.

31. l. 199. Low noise-controlled.

Response: Thanks.

32. l. 220. Differently from Mathematics, in Biology it’s close to impossible to “demonstrate” something as new evidence can change our theories and hypotheses. It’s better to prefer the wording “shown” or synonyms.

Response: Thanks, we have changed it.

33. l. 237. Which kind of alcohol? Ethanol? Pure or diluted? Specify the percentage.

Response: We have added 94% ethanol.

34. l. 252. donors were used.

Response: We have maintained “could be used more than once” as some pairs were used only once.

35. l. 273. Missing space between “X” and “2.5”. Use the “X” symbol consistently (see also l. 278).

Response: Thanks.

36. l. 277. birds used in the trials.

Response: Thanks.

37. l. 290. Any bird preference for.

Response: Thanks.

38. l. 299. Remove “as fixed factor” as you already mentioned it in the methods”.

Response: Thanks, we have removed it.

39. l. 313. See comment 32.

Response: Thanks.

40. l. 326. See comment 32.

Response: Thanks.

41. l. 328. Remove the second “used”.

Response: Thanks, we have corrected it.

42. l. 348. [53], it is still.

Response: Thanks

43. l. 375. Investigations.

Response: Thanks.

44. l. 403. Remove space between “extra-“ and “pair”.

Response: Thanks.

**Response to comment of Reviewer 2**

Reviewer #2: This paper tests for evidence of odour-based discrimination in birds based on MHC class I diversity and similarity. As an understudied area of research, this paper makes a useful contribution to the literature, but I have two main concerns I would like to see addressed before recommending for acceptance: presentation of methods and data, and the focus and scope of the discussion. I discuss in more detail below.

Presentation of methods and data:

It is essential to present at least a summary (e.g. mean +/- SE) of the MHC class I diversity and distances of the stimuli presented to the focal birds. Such data are necessary to evaluate the differences in allelic diversity and genetic similarity of the paired stimuli birds were tested with. For example, it is possible that birds did not respond differentially to donor bird odours because differences in MHC diversity +/or similarity were very small – if that may be the case, it is important to discuss whether a detection capability threshold based on MHC diversity/similarity was likely to have been met or not. Relatedly, if the distances and diversity differences were very small, I recommend the authors analyze the subset of maximally different stimuli pairings (using some reasonable threshold determined a priori) to see if the focal birds presented with maximally different pairings responded differently.

Response: We have provided a table in the supplementary material to show the mean +/- SE and range of the MHC class I diversity and distances of the stimuli presented to the focal birds. In the manuscript, we show the results of a binomial test aimed to analyse whether birds preferred or not the focal bird with more diversity or more dissimilar in MHC-I. However, in a preliminary analysis we performed another binomial test to analyse whether birds could detect amino acid variant numbers of potential mates by using a generalized linear mixed model with binomial errors and a logit link function (GLMM). We modelled the probability that birds chose a particular side of the chamber (as a dichotomous variable: left (yes) vs. right (not)) in relation to the sex and the differences in the number of MHC-I amino acid variants, and in the body condition, between the two scent-donor birds. We included the interaction between the sex and the difference in amino acid variant numbers in the model to test whether males and females differed in their preferences for partners with different numbers of variants of MHC-I amino acids. We also included the pair of donor birds in the model as a random factor to control for the fact that pairs of donors could be used more than once. We included in the initial model a variable reflecting whether the experimental bird left the chamber when we opened the doors or after 1 min as fixed factor. As this variable was not significant (P > 0.49 in all cases), it was removed from the final models. We performed similar analysis to assess whether the difference in functional numbers, or the difference in amino acid distance or in functional distance influenced the choice of birds. Results of this analysis provided the same result, i.e. birds were not exhibiting any preference, so we decided (as previous referees suggested) to use a simpler analysis. We have also provided these analyses in the supplementary material. We have explained in the discussion: “Our results are robust because we used a large sample size and because we used scent-donor birds of similar body condition in each trial to exclude any potential effect of body condition of scent-donor birds in the choice of focal birds. Also, the large sample size used may guarantee that both the MHC-I diversity and similarity distances were likely appropriate for olfactory detection by house sparrows. Furthermore, we performed preliminary statistical analyses using the difference in MHC-I diversity between scent donour partners or the difference in distance between the scent donour partners the focal birds and we observed that the difference in diversity or distance did not explain the choice of birds (see supplementary material). Therefore, we can exclude that differences in MHC-I diversity or similarity were very small and therefore, house sparrows were not able to detect them.”

Given that these were post hoc tests, it seems that these behavioural trials were not originally designed to test MHC-based odour preferences. The authors should be clear about this and state how and when these data were originally used. I don’t necessarily think it’s inappropriate to evaluate the data post hoc, but I think it is important to be clear and up front about this, and to clearly reference any other papers in which these data have been previously used.

Response: In fact, the experiment was designed to test whether house sparrows can use olfaction to assess the number of MHC-I alleles of potential partners. Griggio and collaborators (2011) published the results of a study that showed that females with low allele numbers in MHC-I preferred males with greater allele numbers. So, we aimed to test whether olfaction may be involved in the detection of allele numbers. We captured the birds and determined the allele numbers, using molecular weight, as described in Griggio et al. (2011). Then, we offered the birds the scent of two potential partners differing in the allele numbers that we obtained. Unfortunately, there was a lab mistake in the assessment of allele numbers, that we discovered after performing the behavioural tests, when the breeding season was finished, and we already released the birds. So, we correctly analysed the allele numbers using the molecular weight and tried to publish the results, analysing a posteriori the number of alleles, because the previous analysis was wrong and therefore, the numbers did not match. When we submitted to ms to try to publish it, referees said that the number of alleles calculated using the molecular weight, following Griggio´s paper, was not longer good, so we performed the metabarcoding analysis and we recalculated the number of alleles using the metabarcoding data. In that way, we obtained the allele numbers as well as the functional distances of the conspecifics offered to the focal birds. As we could not use these data when we performed the behavioural experiment, we had to do the analysis a posteriori. We did not explain this in the text, as we think it does not provide relevant information to the readers to understand the ms, but we have now included in the methods that: “For methodological problems, we could not perform the MHC-I characterization before the behavioural tests. The MHC-I characterization was done a posteriori and therefore, we could not pair scent donour birds in relation to their MHC-I diversity or dissimilarity to focal birds.”

Focus and scope of discussion:
Related to the methods and study design, the authors should discuss limitations of the study. For example, with respect to whether the MHC diversity and similarity distances were likely appropriate/sufficient for detection by house sparrows.

Response: Please, see previous response related to this issue. We have added this explanation to the discussion: “The absence of odour-based preference for more diverse or more dissimilar potential partners could hardly be explained by a lack of olfactory abilities in house sparrows because house sparrows are known to use olfaction in social contexts [Fracasso et al. 2019], so a low olfactory capability is also not likely to explain this lack of preference. Our results are robust because we used a large sample size and because we used scent-donor birds of similar body condition in each trial to exclude any potential effect of body condition of scent-donor birds in the choice of focal birds. Also, the large sample size used may guarantee that both the MHC diversity and similarity distances were likely appropriate for olfactory detection by house sparrows. We performed preliminary statistical analysis using the difference in MHC-I diversity between scent donour partners or the difference in distance between the scent donour partners the focal birds and we observed that the difference in diversity or distance did not explain the choice of birds (see supplementary material). Therefore, we can exclude that differences in MHC-I diversity or similarity were very small and that, house sparrows were not able to detect them.”

Currently, the authors spend a lot of time discussing the validity of using first choice, and of tapping on the maze after 1 min to force a choice, but they do not discuss the possibility that this method may not be as appropriate for passerines or sparrows in particular, compared to other species in which first choice is more commonly used, such as seabirds, which may reasonably be expected to behave differently in the test chamber than sparrows do. The authors argue that many studies use this method, but they do not point out that at least as many studies (if not more) use cumulative time spent with a sample type, also with good success (especially for passerines).

Response: We have tried to explain in detail the validity of using first choice, also for Passeriformes, and the limitations of this measure. However, we used live birds as scent donour sources, and therefore, it was not possible for us to assess the time they spent close to the stimuli. We have explained this possibility in the discussion: “Another possibility to explain the lack of preference observed in our study is that a methodological artifact may have masked a preference. In the study with song sparrows, Grieves and collaborators analysed the percentage of time spent close to the two stimuli after 5 minutes from the beginning of the trial. Neither the first choice nor the preference of birds during the first 5 minutes of the trial was reported. We in contrast, analysed the first choice of birds after 5 minutes of exposure. The validity of first choice as a measure of the interest of birds, in particular chemical stimuli, has been previously shown [50, 51, 54, 55, 56], including MHC related scents [15]. However, first choice is a good measure of the spontaneous interest of an animal to a particular cue, whereas time spent close to the stimulus [57] may rather be related to the behaviour shown later on in the series of events to a certain cue. Using life birds as scent donors, the first choice was a valid measure of the response of birds to scent in our study. However, to analyse whether the lack of preferences for the scent of most MHC-I diverse or dissimilar conspecifics is maintained over time, more studies are needed to assess the subsequent response of birds to the scent of potential partners, for instance using uropygial gland secretions as scent sources.”

Finally, there is no discussion of how the timing of the experiment and the physiological condition of the birds could have impacted results or predictions of focal bird preference. In the Leclaire study referenced, birds were incubating and may have preferences related to this, whereas in the Grieves study birds were in breeding condition and may have been motivated differently than in the Leclaire study. This should be included in the discussion alongside consideration of the breeding condition of the birds used in the author’s present study and how this may have affected results and/or predictions/expected outcomes.

Response: We explained in the methods section that we performed the experimental study during the mating period, i.e., during the breeding period with birds that were not paired with birds from the opposite sex and that were not laying or incubating, so they may be in breeding conditions. We now have remarked this point the discussion: “Differences between our results with house sparrows and those with blue petrels can be due to differences in the breeding conditions of birds between those studies, because Leclaire and collaborators tested blue petrels that were incubating [15] and therefore, not searching for a partner. In contrast, we performed the experiment during the breeding period with birds that were separated by sex, and therefore, they were not paired or incubating, so, they may be interested in searching for a partner. Differences between our results and those obtained with song sparrows cannot be attributed to differences in the breeding condition of birds, as in both studies birds were tested during the breeding period and were not incubating.”

Detailed comments:

Line 86: why would MHC I be related to bacterial loads?

Response: We do not know why the authors obtained that result, it may have been an unexpected finding. We have removed the reference.

Lines 93-101: odd to name authors of one study but not the other, and name the study species in one study but not the other. Suggest rephrasing both sentences for consistency of results presentation. It is also worth explaining here or in the discussion the different predictions that can reasonably be made for each sex at different breeding stages (such as during mate choice and pairing vs during incubation) or different predictions that might be made for different taxonomic groups (seabirds vs songbirds) that may differ in level of parental care and investment between the sexes (sex roles, timing of care/investment for each sex, etc).

Response: We have modified the paragraph to be consistent in the use of author names and species names. We have also specified that: “In this study we have experimentally explored whether olfactory signals play a role in the preference for potential partners with greater MHC-I dissimilarity and/or diversity in house sparrows, *Passer domesticus*, during the mating period.”

Line 101: results of Leclaire and Grieves are conflicting but are they controversial? Perhaps not, especially when considering time in breeding cycle as described above.
Response: “Thanks, we meant conflicting, we have rephrased”

Line 115: MHC I, II, or both?

Response: We have now added MHC-I.

Line 120: high or intermediate MHC diversity

Response: Thanks, we have added intermediate.

Line 133: when was the genotyping performed? Birds were captured Feb-Mar 2015 but it’s not clear when genotyping was done in relation to when the behavioural trials were conducted.

Response: We have explained that: “For methodological problems, we could not perform the MHC characterization before the behavioural tests. The MHC characterization was done a posteriori and therefore, we could not pair scent donour birds in relation to their MHC-I diversity or dissimilarity to focal birds.”

Line 136: given that all birds were initially allowed to interact in a group aviary, could it be that mate choice had already taken place, or preferences have otherwise been established? Should discuss whether/how this may have impacted results. Was any evidence of courtship or breeding activity observed during the time birds were group housed?
Response: We have now tried to explain better that birds were located in aviaries and separated by sex, i.e. there were aviaries containing only males and aviaries containing only females, therefore, no reproductive activity could be performed by birds. We have commented on this in the discussion: “Previous experience with scent donour birds may have masked a preference for MHC-I diverse or dissimilar partners. However, we captured birds during February and March and kept females separated from males. Birds were separated by sex in different aviaries, to avoid establishing any interaction with potential partners that may have affected olfactory preferences. Therefore, neither the timing of the experiment, previous interactions during the breeding period or the physiological condition of the birds may explain the lack of preference for MHC-I dissimilar potential partners.”.

Line 143: include primer references/original source for primer development

Response: We have added references, where the primers were designed and modified.

Line 144: please describe what is meant by 'illumina sequencing primer sequences' in more detail so methods can be evaluated.

Response: These Illumina primers (adaptors) are used for allowing the fragments to attach to the cell flow. Now it is explained in the text.

Line 148: please explain how index sequences differ from illumina sequencing primer sequences and why these were attached in a separate round of PCR

Response: These indexes are short sequences (usually around 6 base pairs combinations), unique for each specimen (now indicated in the main text). Two-step PCR is a convenient method to generate amplicon libraries for Illumina sequencing, as it was shown in multiple studies (see for instance, Cruaud et al. 2017. Scientific Reports 7: 41948).

Line 149: demultiplexing?

Response: We are referring here to the possibility of analysing multiple specimens together. The demultiplexing step is posterior.

Line 158: what criteria were used to set this 1% threshold? Overall, more information on sequence data processing methods is needed to properly evaluate these methods. The only reference given is 10 years old and, given the rapidly changing nature of data handling methods in this field, it is essential to ensure the most up to date and appropriate methods are being used for this type of data.

Response: The 1% threshold is still adequate as a first filter to eliminate artefactual alleles. Studies such as Drake et al. (2022) (now cited) support this threshold. We have also added the reference of Razali et al. (2017), which explains the cleaning of the raw data in detail.

Line 159-160: rarefaction is not appropriate for these data (see McMurdie, P.J. and Holmes, S., 2014. Waste not, want not: why rarefying microbiome data is inadmissible. PLoS computational biology, 10(4), p.e1003531.). In Strandh et al. 2012, this 200 cutoff was based on inclusion of technical duplicates, which his reliable only within the sequencing run being evaluated. Were technical duplicates included in this study? If so, this procedure must be described and explained. If not, this method is inappropriate and should not be used. Instead, please update with more recent/current methods.

Response: We did not use duplicates in our data, but we counted on blanks. The number of reads per sample obtained indicated that 200 reads could represent spurious results. For that reason, we have considered this figure as adequate.

Line 169-170: JTT SMS explain/write out in full on first mention

Response: JTT stands for the initials of the authors' names who described this model. We have added the full name and the reference. We have also indicated the meaning of SMS.

Line 201: was the light turned on or was it kept dark throughout the trial?

Response: We have explained that: “Donor birds were kept in darkness during the whole trial duration and reduced space, preventing them from moving or calling.”

Line 217: was experimenter present the entire time? Was experimenter visible to the focal / test birds? Was the test chamber opaque or transparent?
Response: The experimenter was present during the entire trial, but not visible/audible to the focal bird, as the chamber was opaque to maintain birds in darkness.

Line 224: admittedly, I struggle to accept this method as being appropriate... Regardless, it is important to present a summary of how many birds was this done for. A 1 min trial duration seems very short. Many similar studies allow 15 min for birds to choose, even those scoring first choice. I recognize you cannot change this now, but in future, why not use a longer trial duration and evaluate both first choice and choice / time?
Response: We have now explained in the methods that: “68 birds made their choice within less than 1 minute after opening the doors and 33 birds afterwards.” Only one minute may seem to be a short time but, in preliminary trials, we left birds for longer during trial duration and in some cases birds would not move for more than 30 minutes. So, it seems that most birds chose as soon as doors were opened but the ones that did not choose at that moment, spent a long time before moving. Previous results showed that knocking at the door did not influence bird choice, likely because the initial choice of birds was merely reenforced but nor influenced (Amo et al. 2012a,b, 2015). Results of this study showed that knocking at the door did not affect bird choice. Therefore, for ethical reasons, we decided not to unnecessarily increase the time of birds inside the chamber, because not only focal birds were in the chamber, but also scent donour birds.

Line 232: why were only 120 of the original 151 birds tested?

Response: The rest of birds were used in a different behavioural experiment.

Line 235: please include discussion of the side bias exhibited by test birds. How often was the more vs less diverse and more vs less similar individual placed in the left or right side of the olfactometry chamber?

Response: We have explained in more detail that: “Focal birds made 61 % of their first choices to the left and 39 % of the choices to the right side. There were not significant differences in the number of scent donour birds more diverse or more dissimilar between those located in the right side or in the left side of the aviary (GLZM, *P* > 0.30 in all cases). In relation to the amino acid diversity, in 47 trials the most diverse bird was located in the right side of the olfactometry chamber and in 50 trials in the left. In relation to the functional diversity, in 49 trials the most diverse bird was in the right side and in 47 trials in the left. In relation to amino acid distance, the most dissimilar bird was in the right side in 56 trials and in the left in 63 trials. In relation to the functional distance, in 54 trials the most dissimilar bird was in the right side and in 65 trials in the left.”

Line 237: what type and % of alcohol was used?

Response: We have explained that we used “94% ethanol”

Line 266: what were the distances? How was it determined that this was a detectable threshold or that differences in allelic diversity and genetic similarity were otherwise reasonable? Must present data on what MHC distance and allelic diversity pairings were used.
Response: We have provided this data in supplementary material. Please, see the explanation below about the statistical analysis using differences in diversity and distance to explain the bird choice.

Line 286: these trials appear to have been used for multiple experiments/to test multiple questions post hoc. If so, this should be clearly stated somewhere in the methods.

Response: As we explained before, we did not use these trials in other experiment nor to test multiple questions post hoc. We have now explained that: “Due to methodological problems, we could not perform the MHC characterization before the behavioural tests. The MHC characterization was done a posteriori and therefore, we could not pair scent donour birds in relation to their MHC-I diversity or dissimilarity to focal birds.”

Line 316-321: again it's odd presentation to name author in one study and not the other, suggest rephrase so both are consistent.

Response: Thanks, we have corrected it.

Line 321: song sparrows are relatively asocial, blue petrels relatively social, and house sparrows relatively social, but all species may differ in level of sociality during breeding – please discuss how you think sociality may be involved.

Response: Despite that the three species may differ in sociality during the breeding period, we hardly believe that differences in sociality may explain the lack of preference for MHC-I dissimilar partners in our study. We have tried to improve the discussion to implement this potential explanation in our results.

Line 326: many of these studies set a maximum trial duration of 15 min, presumably to allow time for the birds to choose on their own. The present study seems to allow the birds only 1 min, followed by tapping on the test chamber to force the birds to move, which while use in prior studies of this group. The potential impacts of this should be discussed.

Response: Birds were exposed for 5 minutes to the scents, not only one minute. Therefore, if they did not choose during the first minute, the trial duration was extended to 6 minutes in total. Despite that most birds chose within the first minute, we included a variable reflecting whether birds chose within the first minute or after and it did not influence the results.

Line 327: but what MHC differences were used in terms of both diversity and dissimilarity? Again, this information needs to be provided.

Response: We have provided in the supplementary material the means, the data, as well as the analysis of the choice of birds in relation to both the differences in diversity and dissimilarity.

Line 331-341: this would be a good place to discuss the results in terms of the relative differences in MHC I diversity and similarity of the stimuli, and how this may have affected the results.

Response: We have added a paragraph in the discussion. Please, see lines 272-282.

Line 342: returning back to a justification of the first choice method is jarring here; what does this have to do with future work or using preen oil instead of whole body odour? Why would preen oil be superior to use of whole body odour? Suggest restructuring the relevant paragraphs for clarity/flow.

Response: We have tried to clarify it: “Using life birds as scent donors, assessing first choice is a valid measure of the response of birds to scent in our study, because we had to ensure scent-donor birds remain in silent during the trial to avoid acoustic cues that may have affected focal bird choice. However, further studies are needed to assess whether the lack of preference for the scent of most MHC-I diverse or dissimilar conspecifics is maintained over time, for instance using uropygial gland secretions as scent sources.”

Line 347: potential mechanisms for scent-cues of MHC genotype would be better placed in the introduction to set up the study rationale. Since the study did not find evidence for odour choice, I don’t think this discussion of potential mechanisms needs to be here.

Response: Thanks, we have tried to improve this paragraph. We have maintained it in the discussion as we argue that it is needed to perform additional research to examine the mechanisms that may allow MHC-I discrimination in birds.

Line 353: birds lack a VNO – what is the relevance of including this information here?

Response: We have now tried to better explained this.

Line 354: would MHC I be expected to be involved in influencing bacterial profiles?

Response: Thanks, we have tried to clarify.

Lines 357-360: please put this information in the context of the present study.

Response: We have tried to improve the whole paragraph to put the information in the context of our study.

Line 374: a lot of time is spent early in the discussion justifying the use of first choice, but now seems to conclude by saying that different methods should be used - what methods should be used/are recommended? Isn't it worth suggesting that choice over time (cumulative time spent with stimulus) might be a good approach to consider for this type of research in songbirds? First choice has been used successfully in several studies, but so has cumulative time spent, and this should be acknowledged and evaluated. Overall, I think the discussion should be reorganized and more tightly focused around the research question and study results.

Response: Thanks, we have tried to improve the discussion of our results.
