# Supplementary material for: House sparrows do not exhibit a preference for the scent of potential partners with different MHC-I diversity and genetic distances
Source: PLoS One. 2022 Dec 21;17(12):e0278892. doi: 10.1371/journal.pone.0278892 (PMC9770374; doi:10.1371/journal.pone.0278892)
Supplement: Attachment — Submitted filename: Response to referees.docx [file pone.0278892.s008.docx]

**Response to comments of editor, Dr. Ruiz-Rodriguez,**

In the manuscript PONE-D-22-00952R2, authors have made a great effort to improve the quality and clarity of the manuscript. They answered in detail every concern of the reviewers, and performed the requested changes. I appreciate that finally they present the analysis of genetic distances as continuous and categorical variables.

Response: We would like to thank you and the referees for your comments that have substantially improved our ms.

This manuscript is ready to be published in Plos One, but first, I would like to suggest some minor changes:

L178: include some explanation after “and”. E.g. “… of our reads and previous research (40)).

Response: Thanks, we have added “and previous research”.

L384: Remove the comma after ).

Response: Thanks, we have removed the comma.

L430: I think that there is a mistake, given that the preference was for the left side of the chamber.

Response: Thank you for taking notice of the mistake. In fact, we have realized that the mistake was in the Result Section, as preference was for the right size of the chamber. We have corrected in lines 382-384: “However, we found a general side bias impacting the analysis of the bird’s choice with more birds choosing the right side of the chamber (*Z* = -0.83, *P* = 0.03, and *Z* = -2.23, *P* = 0.03, respectively).”.

L434: Is there any reference to support that the laterality does not affect the olfactory preferences of birds?

Response: To our knowledge, there is no previous evidence that brain laterality affects olfactory preferences of birds. We have rephrased the paragraph (lines 430-435): “We found a slight preference for the right side of the chamber that may be attributed to hemispherical asymmetry. House sparrows exhibited laterality in the brain towards the right, as previously reported in other sparrow species, such as American tree sparrows (*Spizella arborea*) [67]. Our results suggest that this laterality does not seem to affect olfactory preferences of focal birds. However, further studies are needed to explore olfactory laterality in avian species.”
